# Effects of Essential Oil Citral on the Growth, Mycotoxin Biosynthesis and Transcriptomic Profile of *Alternaria alternata*

**DOI:** 10.3390/toxins11100553

**Published:** 2019-09-20

**Authors:** Liuqing Wang, Nan Jiang, Duo Wang, Meng Wang

**Affiliations:** 1 Beijing Research Center for Agricultural Standards and Testing, No. 9 Middle Road of Shuguanghuayuan, Haidian District, Beijing 100097, China; wanglq@brcast.org.cn (L.W.); jiangn@brcast.org.cn (N.J.); wangduo@brcast.org.cn (D.W.); 2 Laboratory of Quality & Safety Risk Assessment for Agro-products (Beijing), Ministry of Agriculture and Rural Affairs, No. 9 Middle Road of Shuguanghuayuan, Haidian District, Beijing 100097, China

**Keywords:** *Alternaria alternata*, mycotoxin, alternariol, essential oil, cell integrity, oxidative stress

## Abstract

*Alternaria alternata* is a critical phytopathogen that causes foodborne spoilage and produces a polyketide mycotoxin, alternariol (AOH), and its derivative, alternariol monomethyl ether (AME). In this study, the inhibitory effects of the essential oil citral on the fungal growth and mycotoxin production of *A. alternata* were evaluated. Our findings indicated that 0.25 μL/mL (222.5 μg/mL) of citral completely suppressed mycelial growth as the minimum inhibitory concentration (MIC). Moreover, the 1/2MIC of citral could inhibit more than 97% of the mycotoxin amount. Transcriptomic profiling was performed by comparative RNA-Seq analysis of *A. alternata* with or without citral treatment. Out of a total of 1334 differentially expressed genes (DEGs), 621 up-regulated and 713 down-regulated genes were identified under citral stress conditions. Numerous DEGs for cell survival, involved in ribosome and nucleolus biogenesis, RNA processing and metabolic processes, and protein processing, were highly expressed in response to citral. However, a number of DEGs responsible for the metabolism of several carbohydrates and amino acids, sulfate and glutathione metabolism, the metabolism of xenobiotics and transporter activity were significantly more likely to be down-regulated. Citral induced the disturbance of cell integrity through the disorder of gene expression, which was further confirmed by the fact that exposure to citral caused irreversibly deleterious disruption of fungal spores and the inhibition of ergosterol biosynthesis. Citral perturbed the balance of oxidative stress, which was likewise verified by a reduction of total antioxidative capacity. In addition, citral was able to modulate the down-regulation of mycotoxin biosynthetic genes, including *pksI* and *omtI*. The results provide new insights for exploring inhibitory mechanisms and indicate citral as a potential antifungal and antimytoxigenic alternative for cereal storage.

## 1. Introduction

*Alternaria alternata* is a widespread phytopathogen, causing serious foodborne spoilage and producing a variety of mycotoxins that are detrimental to human and animal health through food chains. *Alternaria* mycotoxins have gradually been paid more attention in relation to public health, as suggested by the European Food Safety Authority (EFSA) [1]. The major *Alternaria* mycotoxins are alternariol (AOH), alternariol monomethyl ether (AME), altenuene, tenuazonic acid, and altertoxins [2,3]. AOH and AME are two of the most frequent contaminants in food and feedstuffs derived from cereals, fruits and vegetables [2,4]. AOH and AME exert genotoxicity and mutagenicity as topoisomerase poison inducing DNA strand breaks [5], and further they are a possible factor in human oesophageal cancer in China [6,7]. Besides, they possess cytotoxicity as well as reproductive and developmental toxicity [4]. In addition, *A. alternata* is regarded as a critically common cause of allergic rhinitis and atopic asthma owing to the production of *Alternaria* allergens, such as the major allergen, Alt a 1 [8,9].

To date, the AOH and AME biosynthetic pathway has been unraveled, and a polyketide gene cluster is responsible for their production in the genome of *A. alternata* [10]. Among the clustered genes, *pksI* encoding for polyketide synthase was sufficient for AOH biosynthesis, which was verified by gene disruption in *A. alternata* and heterologous expression in *Aspergillus oryzae*. *OmtI* encoding for O-methyl transferase was responsible for AME formation from the methyl ether of AOH. Except for *omtI*, another three clustered genes, encoding a mono-oxygenase (*moxI*), a short-chain dehydrogenase (*sdrI*) and an estradiol dioxygenase (*doxI*), were also involved in AOH modification. In this cluster, a fungal specific transcriptional factor encoded by *aohR* positively regulated the expression of other clustered genes. In addition, *pksI* homologous gene, *SnPKS19*, was illustrated to be involved in AOH biosynthesis in the wheat pathogen *Parastagonospora nodorum*, and likewise this was further proved by heterologous expression in *A. nidulans* [11]. Moreover, AOH and AME demonstrated potential phytotoxic activity as pathogenicity factors in their study. AOH facilitated the infection and colonization of *A. alternata* on tomato, citrus and apple by the addition of AOH to *pksI*-deletion mutant, but the chemical alone exhibited no phytotoxin in wheat leaves and seed germination [10,11]. Correspondingly, AME enabled the inhibition of photosynthetic electron transport in extracted spinach chloroplasts [12].

Essential oils are easily volatile aromatic compounds extracted from plant material, including terpenes, aldehydes, esters, etc. They are environmentally friendly and are employed as ingredients in drugs, cosmetics, etc. The components of essential oils, such as citral, cinnamaldehyde, eugenol and thymol, exhibit strong antifungal properties as well as the inhibition of mycotoxin production [13,14,15]. Among them, citral has broad-spectrum inhibitory effects against various plant pathogens, including *A. solani* [16], *Penicillium italicum* [17,18,19], *P. expansum* [20], *A. flavus*, *Fusarium moniliforme*, etc. [21]. Furthermore, citral displayed cytotoxicity against *P. italicum* by affecting mitochondrial dysfunction, damaging membranes and inhibiting ergosterol biosynthesis in previous studies [17,18,19]. Moreover, the combination of citral and cinnamaldehyde highly suppressed the growth and patulin production of *P. expansum* by oxidative damage and down-regulation of the patulin biosynthetic pathway [20]. In addition to the antifungal activity of *A. flavus*, citral exerted antiaflatoxigenic activity by modulating aflatoxin biosynthetic gene expression [22,23].

Citral revealed fungitoxic activity on *A. alternata* by a paper disc agar diffusion assay, as suggested by Kishore et al. [21]. However, until now, citral’s mode of action in repressing growth and mycotoxin production in *A. alternata* has not yet been elucidated. In this study, the inhibitory effects of citral on fungal growth and mycotoxin biosynthesis were evaluated in *A. alternata*. Furthermore, great efforts were made to uncover the potential mechanisms through a comprehensive and systematical view by RNA-Seq among samples with or without citral treatment. Our findings offer promising insights into citral’s application for the control of fungal infection and mycotoxin contamination.

## 2. Results

### 2.1. Inhibitory Effects on Mycelial Growth, Spore Germination and Mycotoxin Production

**Figure 1:**
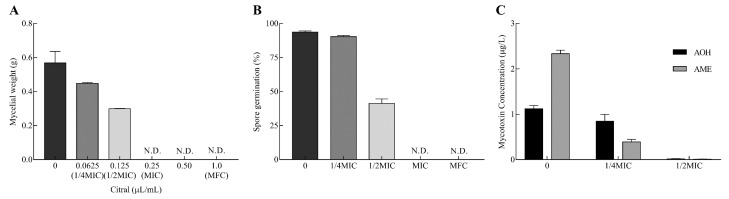
Citral affects mycelial weight, spore germination and mycotoxin production of *A. alternata*. (**A**) The mycelial weight of *A. alternata* was determined after drying under serial concentrations of citral. Minimum inhibitory concentration (MIC): 0.25 μL/mL (222.5 μg/mL); minimum fungicidal concentration (MFC): 1.0 μL/mL (890 μg/mL). (**B**) The germination rate of *A. alternata* spores exposed to citral. (**C**) The determination of mycotoxins, alternariol (AOH) and alternariol monomethyl ether (AME) produced by *A. alternata* in response to citral. The results are illustrated as mean ± SEM (*n* = 3).

The inhibitory effects of citral against *A. alternata* are displayed in Figure 1. The mycelial growth was significantly inhibited in a dose-dependent manner (Figure 1A). The inhibition percentage rose from 21.3% to 47.5% with the increase in citral concentration from 0.0625 to 0.125 μL/mL (from 55.625 to 111.25 μg/mL). The mycelial growth of *A. alternata* was completely suppressed at 0.25 μL/mL (222.5 μg/mL) as the minimum inhibitory concentration (MIC). Moreover, the minimum fungicidal concentration (MFC) of citral was determined to be 1.0 μL/mL (890 μg/mL) by the observation of no mycelial growth on the citral free culture after citral treatment.

Likewise, spore germination was markedly repressed in response to different concentrations of citral (Figure 1B). The presence of spore germination was almost entirely observed in the control by light microscopy, while the absence of spore germination was observed at the MIC and MFC of citral. Therefore, citral revealed outstanding antifungal properties against mycelial growth and spore germination in *A. alternata*.

Citral obviously repressed AOH and AME production in *A. alternata* (Figure 1C). At 1/4MIC of citral, AOH concentration did not sharply reduce in *A. alternata* (*p* = 0.19). However, AOH was hardly biosynthesized by *A. alternata* and declined by 98.6% at 1/2MIC. Citral was markedly effective in resisting against AME production at 1/4MIC and 1/2MIC. The inhibition rates exhibited were 83.3% and 99.6%, respectively, compared with the control. In connection with mycelial growth, the inhibition of mycelial growth was not the only cause of the reduction of the mycotoxin amount.

### 2.2. Global Analysis of Transcriptomic Profile

**Figure 2:**
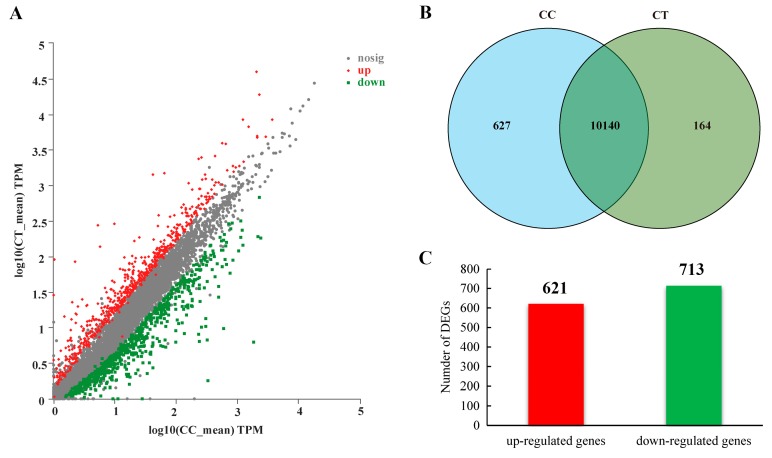
Summary of RNA-Seq analysis. (**A**) Statistical scatter diagram of differential gene expression. Compared to the control of *A. alternata* with no citral (CC), the pattern of gene expression was indicated as up-regulation (up), down-regulation (down) and no significant differential expression (nosig) under the condition of citral treatment (CT). TPM: transcripts per million reads. (**B**) Venn diagram of the transcribed genes between citral-treated and untreated samples. (**C**) Number of differentially expressed genes (DEGs) in *A. alternata* exposed to citral stress. Out of 1334 DEGs, 621 genes were highly up-regulated, and 713 genes were markedly down-regulated.

Comparative transcriptome analysis of *A. alternata* was performed to systematically discover the potential antifungal and antimycotoxigenic mechanisms between the control and 1/2MIC citral treatment. The total statistics of the RNA-Seq data are summarized in Table S1. More than 48 million clean reads were obtained after removing adaptor sequences, low-quality reads, sequences with 10% higher ambiguous bases, and over-short sequences. The average rate of total mapped reads was about 87.4% after the alignment with the sequence of the reference genome. The results of the correlation between biological replicates were higher than 0.96, which was performed by Pearson’s correlation coefficient based on the expression matrix. Out of 13,761 genes in total, Veen analysis showed that there were 10,767 transcribed genes in the control and 10,304 expressed genes in the 1/2MIC citral treatment (Figure 2A,B). It was shown that 627 unique genes were only transcribed in the control, and 164 genes were solely expressed under the citral condition.

The analyses of RNA-Seq data involved great efforts to explore the potential differential genes and pathways in *A. alternata* under citral stress. Differentially expressed genes (DEGs) were analyzed and are revealed in Table S2 based on the absolute value of fold change (FC) at ≥2 and false discovery rate (FDR) at < 0.05. In summary, there were 1334 DEGs found within the two groups of citral-treated and untreated fungi, with 621 (47%) up-regulated and 713 (53%) down-regulated DEGs compared to the control (Figure 2A,C).

### 2.3. Functional Analysis of DEGs

**Figure 3:**
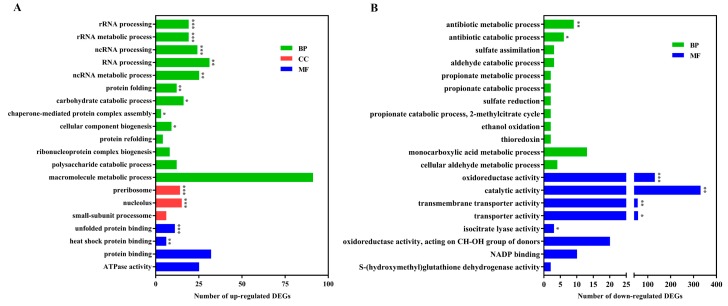
Gene ontology (GO) enrichment analysis of differentially expressed genes (DEGs). Top 20 results of the highest enrichment level were separately obtained from the analysis of up-regulated DEGs (**A**) and down-regulated DEGs (**B**). ***: false discovery rate (FDR) < 0.001; **: FDR < 0.01; *: FDR < 0.05.

To further characterize the functional differences and relationships of DEGs, they were excavated by Gene Ontology (GO) enrichment and Kyoto Encyclopedia of Genes and Genomes (KEGG) pathway enrichment analyses. Based on the significant results of the GO enrichment analysis, there were obvious differences between GO enrichment analyses separately performed based on the up-regulated or down-regulated DEGs (Figure 3). The up-regulated DEGs in association with the processes of ribosome formation and protein processing were highly represented, including rRNA processing and metabolic processes, ncRNA processing and metabolic processes, RNA processing, preribosome, protein folding and unfolded protein binding, which are essential for fundamental fungal survival during exposure to stress. Nevertheless, the down-regulated DEGs were significantly enriched in oxidoreductase activity, antibiotic metabolic processes, catalytic activity, and transporter activity. The result of gene expression related with cellular RNA behavior was a little different from *P. digitatum* in response to citral stress over a short time [17]. This might reveal that there are some differences in antifungal mechanisms for different fungal species under long- or short-term chemical treatment.

**Figure 4:**
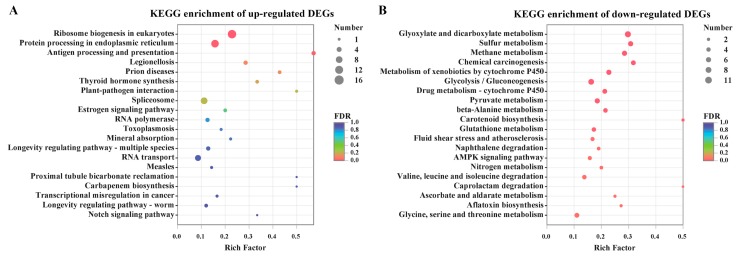
Kyoto Encyclopedia of Genes and Genomes (KEGG) metabolic pathway enrichment analysis of differentially expressed genes (DEGs). Top 20 results of the highest enrichment level were acquired from the individual analysis of up-regulated DEGs (**A**) or down-regulated DEGs (**B**).

To uncover the metabolic pathway of these DEGs, they were mapped into the KEGG pathway database. Ribosome biogenesis in eukaryotes and protein processing in endoplasmic reticulum were the most significant enrichment of pathways in the up-regulated DEGs (Figure 4). However, there were a large number of categories in relation to carbohydrate metabolism, energy metabolism and xenobiotics biodegradation, which were highly represented in the down-regulated DEGs. To a certain extent, a number of fungal processes for primary metabolism, especially carbohydrate and energy metabolism including glyoxylate and dicarboxylate metabolism, pyruvate metabolism glycolysis/gluconeogenesis and methane metabolism, were seriously hampered by citral stress. Correspondingly, among these genes, 11 DEGs were shown to be down-regulated in pyruvate metabolism, and eight DEGs were inhibited in the glycolysis/gluconeogenesis pathway by citral. In addition, the processes of nitrogen metabolism and amino acid metabolism were partially repressed under the citral condition.

### 2.4. Genes Responsible for Cell Integrity

**Figure 5:**
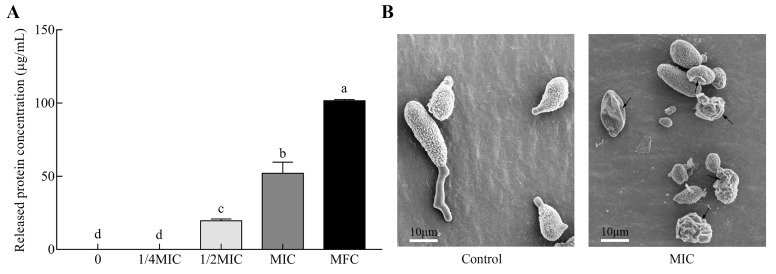
Permeability and morphological alteration of *A. alternata* in response to citral. (**A**) Intracellular protein leakage of *A. alternata* spores treated with serial concentrations of citral (*n* = 3). MIC: minimum inhibitory concentration; MFC: minimum fungicidal concentration. Significant differences (*p* < 0.05) are indicated by different lowercase letters above the bars. (**B**) Images of fungal spores under the conditions of 0 and MIC of citral by scanning electron microscopy.

Cell integrity is essential for fungal survival when exposed to chemical stress. The fungal disruption induces the efflux of cytoplasmic constituents. In this study, the permeability of cytoplasmic constituents was reflected by the leakage of intracellular proteins released from fungal spores of *A. alternata* treated with different concentrations of citral (Figure 5A). Protein release was positively dose-dependent with citral concentration. At the MFC (1.0 μL/mL; 890 μg/mL) of citral, the released protein concentration significantly increased up to 101.7 μg/mL. This showed that cell damage was much more severe in a concentration-dependent way. The permeability was coincident with the disruption of fungal spores observed by microscopic morphology (Figure 5B). The conidia revealed a massive distortion of morphological structure and abnormal cell shrinkage under the MIC of citral. Even worse, there were several completely disrupted spores that occurred in the citral-treated solution.

**Figure 6:**
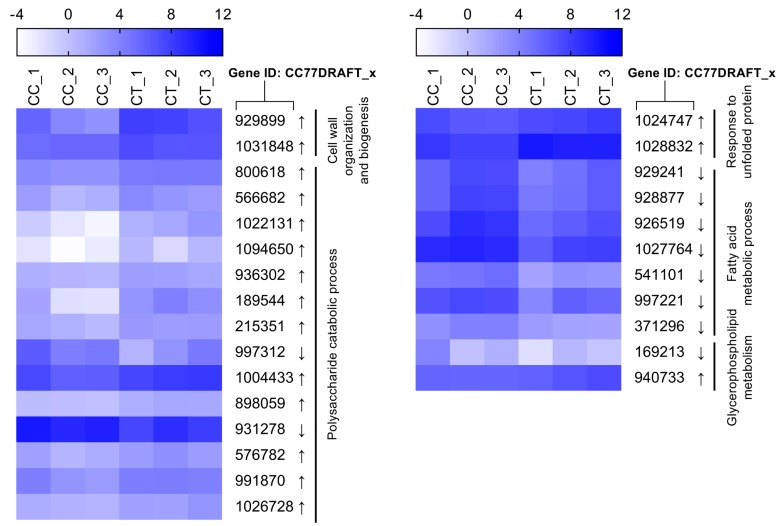
Comparative expression level of DEGs potentially involved in cell integrity. Gene transcriptional values are indicated as log_2_ TPM (transcripts per million reads) for each biological replicate of *A. alternata* with (CT) or without (CC) citral treatment. Arrows next to the gene IDs are used to represent up-regulated (↑) and down-regulated (↓) DEGs.

The fungal cell wall firstly senses the pressure of toxic compounds in the external environment as the outermost defensive line. Two DEGs in relation with cell wall biogenesis and organization were highly up-regulated 3.162- and 6.321-fold in response to citral (Figure 6). Polysaccharides are the principal components for cell wall structure. Of seven DEGs responsible for the polysaccharide catabolic process, except NADP-dependent mannitol dehydrogenase encoding genes, six DEGs were overexpressed between 2.574- and 4.109-fold under the citral condition. In addition, the conserved cell wall integrity pathway is affected by the unfolded protein response in filamentous fungi [24]. In this work, the expression of two genes (*CC77DRAFT_1024747* and *CC77DRAFT_1028832*) involved in the response to unfolded protein was disturbed under chemical stress.

In previous studies, essential oils have been proposed to possess antifungal and antimycotoxigenic potency via the disruption of the plasma membrane as a potential target [25,26,27]. Ergosterol amount significantly decreased by 36.6% from 3.3 to 2.1 mg/g in response to citral at 1/2MIC compared to the control (Figure S1). Correspondingly, two genes encoding a C-3 sterol dehydrogenase/C-4 decarboxylase-like protein (*ERG26*; *CC77DRAFT_590852*) and a 3-keto-steroid reductase (*ERG27*; *CC77DRAFT_76456*) were differentially expressed in the pathway of ergosterol biosynthesis. However, while the *ERG26* transcriptional level was lower, *ERG27* was more highly transcribed under the stress condition. Virtually, ERG26 catalysate is the substrate of ERG27. A lower intermediate catalyzed by ERG26 was supplied for the following catalysis during the process of ergosterol biosynthesis, in spite of the higher mRNA level of *ERG27*. This might be the reason why ergosterol production drastically declined after citral treatment. Glycerophospholipid plays an important role in the component of the plasma membrane. Glycerophospholipid metabolism was partially interfered with for the differential expressions of *CC77DRAFT_169213* and *CC77DRAFT_940733*. In addition, fatty acid biosynthesis contributed to the fluidity of plasma membranes, and seven DEGs for fatty acid metabolic processes were significantly down-expressed in response to citral, especially two genes of fatty acid synthase activity (*FAS1*: *CC77DRAFT_928877*; *FAS2*: *CC77DRAFT_929241*).

### 2.5. Genes Related to Stress Response

**Figure 7:**
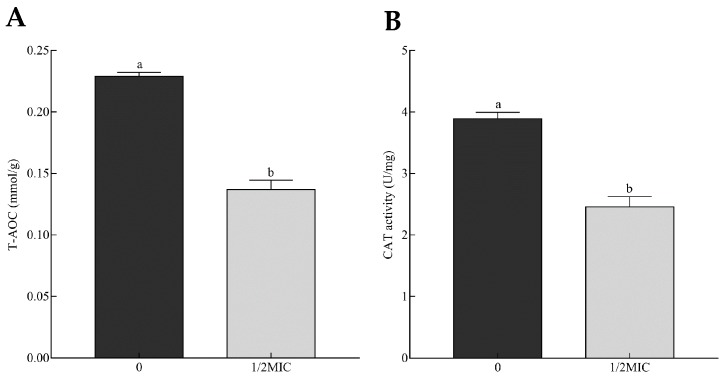
Total antioxidant capacity (**A**) and catalase activity (**B**) of *A. alternata* exposed to 0 and 1/2MIC (minimum inhibitory concentration) of citral. The data are expressed as the mean ± SEM (*n* = 3). Different lowercase letters above the column indicate statistically significant results (*p* < 0.05).

Exposure to citral led to a wide alteration of *A. alternata*’s transcriptomic profile in association with stress response. Essential oils can interfere with the homeostasis of oxidative stress and induce the imbalance of reactive oxygen species (ROS), such as hydrogen peroxide (H_2_O_2_), superoxide (O_2_^−^), and hydroxyl radical (·OH), during the inhibition of mycotoxin-producing fungi [20,28,29]. Total antioxidant capacity (T-AOC) indeed mirrors the level of ROS balance. In this study, the T-AOC of *A. alternata* was reflected by the ferric reducing ability of the Ferric Reducing Ability of Plasma (FRAP) method and significantly reduced from 0.23 to 0.14 mmol/g with the increase in citral concentration (Figure 7A). This reduction could result from the decreasing content of antioxidant materials scavenging ROS. This might lead to ROS instability and, eventually, the damage of cell structure and secondary metabolism. This was the possible cause of cell debris and the mycotoxin reduction of *A. alternata* suppressed by citral.

**Figure 8:**
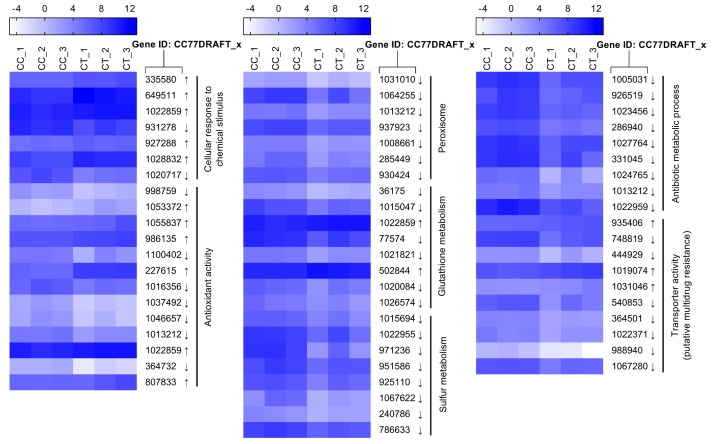
Comparative expression level of DEGs putatively responsible for stress response. Gene expression values are expressed as log_2_ TPM (transcripts per million reads). Arrows represent up-regulated (↑) and down-regulated (↓) DEGs.

Both enzymatic and non-enzymatic systems are developed for maintaining ROS balance, including cellular detoxifying enzymes and reducing substances. Catalase can catalyze H_2_O_2_ to detoxify ROS stress. Two isozymes of catalase (CC77DRAFT_364732 and CC77DRAFT_1013212) were significantly down-regulated. Correspondingly, the activity of catalase decreased by 36.9% compared to the control (Figure 7B). In addition, peroxisomes exhibit multifunctional activities, including the decomposition of ROS [30,31]. In this work, seven DEGs responsible for peroxisome biogenesis were less transcribed under the citral condition (Figure 8). This probably resulted in the dysfunction of peroxisome, which was detrimental to the survival of *A. alternata*. Glutathione metabolism responsible for oxidative balance was enriched in down-regulated DEGs from KEGG analysis. Glutathione S-transferase, belonging to the glutathione system, catalyzes the conjugation between glutathione and many xenobiotic compounds for the reduction of their toxicity [32]. They were down-regulated in *A. alternata* in response to citral, including *CC77DRAFT_36175*, *CC77DRAFT_1015047*, and *CC77DRAFT_1026574*. Additionally, DEGs of oxidoreductase activity might be likewise vital to supply reducing power to protect from the damage of ROS accumulation, but they were significantly enriched in down-regulated DEGs. Moreover, sulfur metabolism, including sulfate assimilation and sulfate reduction, plays practical roles in stress tolerance [33,34]. Sulfur-containing defense compounds, including sulfide, glutathione, and various secondary metabolites, as well as sulfur-rich proteins, are crucial for fungal survival under abiotic stress [33,35]. The transcripts of eight DEGs in relation to sulfur metabolism were all repressed by citral in *A. alternata*. In connection with the results of T-AOC and catalase activity, fungal cells could not remove the accumulation of ROS in a timely manner after citral treatment. This might give rise to the sharp violation of ROS balance, and the disorder of oxidative stress eventually resulted in the disruption of cell structure and the disturbance of mycotoxin biosynthesis.

There are some other defense systems that resist against abiotic stress, such as multidrug resistance [17]. Drug metabolic processes, including antibiotic metabolic processes, may assist in multidrug resistance. The results of GO enrichment demonstrated that these processes were partially impaired by citral. Furthermore, transporters, especially ATP-binding cassette (ABC) transporter and major facilitator superfamily (MFS) transporter, play an important role in the efflux capacity of xenobiotic compounds [36]. Correspondingly, putative multidrug resistance of transporter activity was notably inhibited by the essential oil, which was unfavorable to alleviate the stress in *A. alternata*.

### 2.6. Citral Interferes with the Expression of Genes Responsible for Mycotoxin Biosynthesis

**Figure 9:**
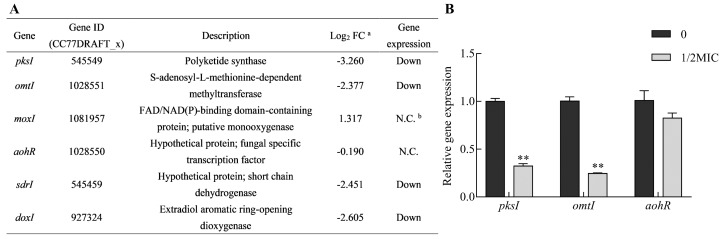
Transcriptional analysis of the clustered genes involved in AOH and AME production. (**A**) Table showing the results of gene expression from the analysis of RNA-Seq. ^a^ FC: fold change; ^b^ N.C.: no significant change in gene expression level. (**B**) Comparative analysis of gene expression by quantitative reverse transcription PCR (qRT-PCR). *PksI*, *omtI* and *aohR* were directly responsible for AOH and AME biosynthesis and regulation in *A. alternata*. The data are presented as the mean ± SEM (*n* = 3). MIC: minimum inhibitory concentration.

The biosynthetic gene cluster responsible for AOH and AME biosynthesis has been elucidated [10]. It was sufficient for AOH formation by *pksI* (*CC77DRAFT_545549*) in *A. alternata* [10,11]. AME is the product of AOH methylation catalyzed by an *omtI* (*CC77DRAFT_1028551*) encoding methyl-transferase. Another three enzymes (moxI, sdrI and doxI) were also involved in AOH modification. Additionally, a GAL4-like Zn(II)2Cys6 transcription factor expressed by *aohR* (*CC77DRAFT_1028550*) in the *pksI*-gene cluster modulated the transcriptional enhancement of the clustered synthase genes. The expression of four clustered genes (*pksI*, *omtI*, *sdrI* and *doxI*) showed down-regulation, as observed from RNA-Seq data, in the citral treatment (Figure 9A). To validate the analyses from RNA-Seq data, three clustered genes (*pksI*, *omtI* and *aohR*) directly involved in AOH and AME biosynthesis and regulation were chosen to be employed for quantitative reverse transcription PCR (qRT-PCR) analysis. The results of these gene expression patterns were similar to the outcomes of transcriptomic analysis (Figure 9A,B). This further demonstrated that citral could modulate the down-regulation of biosynthetic genes, including *pksI* and *omtI*.

## 3. Discussion

*A. alternata* is an important phytopathogen, causing agricultural output losses and playing a tremendous role in food and feed safety due to the production of mycotoxins. Of these mycotoxins, AOH and AME were shown to be two of the most frequently contaminated mycotoxins [2,3]. To manage this contamination, essential oils have been shown to be environmentally friendly alternatives to common antifungal agents, especially in consideration of postharvest contamination of food and feed with *Alternaria* mycotoxins. Citral has been shown to be brilliant in suppressing fungal infection and mycotoxin contamination in *P. expansum* [20], *A. ochraceus* [37] and *Fusaria* [38]. Among them, the potential antifungal and antimycotoxigenic mechanisms were illustrated via transcriptomic profiling in the inhibition of *P. expansum* by the combination of cinnamaldehyde and citral [20]. Nevertheless, the distinction of these two essential oils and the exact action mode of citral alone was still not clearly known in resisting against *P. expansum*. Additionally, essential oil, to a great extent, played different roles in cellular response in various fungal species in previous studies [14]. Therefore, the inhibitory effects and mechanisms of citral suppressing the growth and mycotoxin production of *A. alternata* were uncovered in this study.

Citral exerted highly inhibitory effects on the mycelial growth and mycotoxin production of *A. alternata*. The antifungal efficiency was similar to that of cinnamaldehyde described by Xu et al. [25]. Citral has broad-spectrum antifungal activity, and it was much more effective than eugenol, geraniol, limonene and linalool, impairing *A. alternata* as well as *A. niger*, *A. flavus*, *F. moniliforme*, etc. [21]. Citral exhibited strong fungicide capacity by disrupting cell structure as shown by comparative microscope analysis with or without chemical stress. This antifungal finding can be regarded as similar to former reports on a number of essential oils such as cinnamaldehyde [25,39], eugenol [40], and thymol [41]. In addition, fungicidal activity was further demonstrated by the inhibition of spore germination. This result was consistent with that of *Nepeta rtanjensis* essential oil, which suppressed the elongation of the germ tube and spore germination, observed by light microscopy, as the concentration increased [42,43]. The sharp enhancement of fungal cell permeability was reflected by the abnormal and massive spillage of intracellular soluble proteins from macromolecular cytoplasmic components owing to cell damage by citral. In previous studies, intracellular reducing sugar and protein influenced the cell lysis rate by (*E*)-2-hexenal on *A. flavus* [44] or 7-demethoxytylophorine on *P. italicum* [45]. Accordingly, citral caused spore lysis in a dose-dependent manner from the result of released soluble protein in our study.

Fungal cell integrity is essential to adapt to the stress response caused by toxic compounds. The lipophilic terpenoid intrudes into plasma membranes and causes the disturbance of membrane integrity. Ergosterol is the major component of structural sterols, especially in the fungal cell membrane. The disorder of ergosterol may lead to much more fragility in response to stress, while the marked reduction of ergosterol could result from citral treatment in this work. The disruption of plasma membranes makes fungal cells so much more vulnerable as citral disturbs the exchange of substantial and energy metabolism via plasma membranes. This could be the mechanism of the cytotoxicity of citral against *A. alternata*. In combination with the result of scanning electron microscopy, exposure to the MIC of citral led to seriously deleterious damage to the cell plasma membrane. Furthermore, it would be necessary to carry out further studies to understand the actual relationship between the disruption of plasma membranes and the inhibition of mycotoxin production. Moreover, fatty acid biosynthesis also influences the membrane fluidity and rigidity. Correspondingly, the expression pattern of DEGs involved in the fatty acid biosynthesis pathway was significantly impaired by citral. In a previous study, the other monoterpene, *d*-limonene, induced cytotoxicity on the alteration of the cell wall as the main target but not on the plasma membrane in *Saccharomyces cerevisiae* [46]. However, another monoterpene, α-terpinene, displayed strong antagonistic activity against *S. cerevisiae* and the overexpression of numerous genes in relation to the cell wall and also membrane biogenesis [47]. In our study, the considerable alteration of the cell surface and membrane was eventually revealed by the results of ergosterol content, shrunken and wrinkled cell surfaces and RNA-Seq data under the citral stress in this report.

Fungal oxidative damage has been considered as an essential factor for the antifungal and antimycotoxigenic properties of essential oils [20,28,29]. ROS are inevitably produced by essential oils in response to stress, which can react with intracellular components and lead to chemical damage, such as lipid peroxidation, protein oxidation, etc. Overaccumulation of ROS can eventually cause defective cells and even lethal cells. On the other hand, fungal antioxidative defense systems of ROS stress have been evolved to detoxify ROS, including enzymatic systems, such as catalases and superoxide dismutases, and also non-enzymatic systems, such as glutathione and thioredoxin [32]. ROS exert multiple roles as signaling molecules for fungal growth, development, biosynthesis of secondary metabolites and other cell processes. Consequently, the maintenance of ROS level is critical to the survival of the fungal life cycle. In this work, the total antioxidative capacity of *A. alternata* exposed to citral was markedly reduced. Correspondingly, ROS could accumulate and this could be detrimental to fungal survival under the citral condition. In addition, catalase activity was also demonstrated to be lower after citral treatment through an enzyme activity assay and RNA-Seq data. This would imply that hydrogen peroxide, the substrate of catalase, could be difficult to scavenge in time. These results demonstrated that the balance of ROS level was disturbed after the exposure to citral. In recent studies, similar observations revealed that the antifungal and antimycotoxigenic characteristics of essential oils were highly connected with the induction of ROS formation [28,29,48]. For instance, the inhibitory effects of cinnamaldehyde against mycelia growth and aflatoxin B_1_ could be attributed to the perturbation of redox status [28]. Enriched analysis of DEGs also demonstrated that oxidoreductase activity, glutathione metabolism and sulfur metabolism were overall down-regulated in *A. alternata* in response to citral stress. These cellular processes were closely related to ROS maintenance. Nevertheless, the abnormality of these cellular processes was unfavorable to the survival of *A. alternata* exposed to citral. Interestingly, siderophore was demonstrated to be resistant against oxidative stress [49]. A nonribosomal peptide synthetase encoding gene (*AaNPS6*), involved in siderophore biosynthesis [50], was highly regulated after citral treatment. However, the increase seemed to be inadequate to alleviate the accumulation of oxidative stress and fungal damage in this study, as evidenced by shrunken and wrinkled cell surfaces and the reduction of total antioxidative capacity. On the other hand, the alteration of carbon flow on the biosynthesis of secondary metabolites might be the reason for the marked reduction of AOH and AME. In addition, multidrug resistance facilitates fungal defense against xenobiotic stress. Except for potential drug metabolic processes, fungal ABC and MFS transporter proteins are essential for the efflux of toxic compounds for antifungal drug resistance [36,51]. A number of these functional genes in association with cellular multidrug resistance were down-regulated by citral. This reduction tended to be harmful to fungal survival with exposure to citral.

In this study, it was demonstrated that the repression of biomass was not the only cause of the reduction of AOH and AME production. This could be likewise attributed to the obstacle of mycotoxin biosynthetic genes under the citral condition. *PksI* and *omtI*, involved in mycotoxin biosynthesis, were less transcribed, while the expression of *aohR* encoding a specific transcriptional factor showed a slight decrease but no significant change under citral stress. *PksI* was sufficient for AOH formation in *A. alternata*, which was further confirmed by heterougous expression in *A. oryzae* [10]. In addition, *omtI* encoded a methyl-transferase, catalyzing the transformation from AOH to AME. Mycotoxin production could be significantly reduced by down-regulating the transcriptional level of the two enzymatic genes in this work. Similar results of gene expression were observed in the mycotoxin biosynthetic pathway after essential oil treatment. *AcOTApks* and *acOTAnrps*, directly responsible for ochratoxin A (OTA) biosynthesis, were obviously down-regulated by essential oils such as fennel and cardamom, even though they did not affect the growth of *A. carbonarius* S402 [52]. However, a recent study demonstrated that both the mycelial growth and transcriptional level of *pks* and *nrps* involved in OTA production were down-regulated in *A. ochraceus* fc-1 in response to cinnamaldehyde [39]. This reveals that there exist different inhibitory mechanisms of essential oils suppressing fungal growth and mycotoxin production. Another similar study was conducted, in which the expression of aflatoxin biosynthetic genes, such as *aflD*, *aflM*, *aflO*, *aflP*, and *aflQ*, was suppressed by turmeric essential oil [53]. Interestingly, one transcriptional regulator gene, *aflR*, in the aflatoxin biosynthetic gene cluster was significantly inhibited, while the other regulation enhancer, *aflS*, was observed to have no obvious changes, like *aohR*, in terms of transcriptional level. The expressions of *nor-1* (*aflD*), *ver-1* (*aflM*) and *omt-A* (*aflP*) were likewise repressed in toxigenic *A. parasiticus* exposed to *Zataria multiflora Boiss* essential oil [54,55].

In conclusion, the antifungal and antimycotoxigenic mechanisms of citral on the growth and mycotoxin production of *A. alternata* were unraveled in this study. Citral caused irreversible damage to the spore ultrastructure. In addition, citral was able to disturb oxidative balance, disrupt cell integrity, repress transporter activity and down-regulate biosynthetic genes of AOH and AME, including *pksI* and *omtI*. Additionally, citral, as well as other essential oils like cinnamaldehyde, eugenol, thymol, etc. has been generally recognized as safe and registered for food flavoring in the European Union, regarded as having no toxicity and preservative potential [15,56]. However, citral was also indicated as a contact irritant and a contact allergen. Therefore, it is more applicable for unprocessed food raw materials, such as cereal storage. Besides, there is the great challenge of its strong fragrance even at relatively low concentrations. Encapsulation could potentially provide a novel strategy for its usage to minimize the organoleptic impact, especially nanoencapsulation. In general, in view of its environmental friendliness and highly fungicidal characteristics, citral may be a potentially promising alternative as a chemical fungicide for cereal storage.

## 4. Materials and Methods

### 4.1. Chemicals, Strain and Culture Conditions

Mycotoxin standards of AOH and AME were purchased from Romer Labs (Newark, DE, USA). Ergosterol was obtained from Sigma-Aldrich (St. Louis, MO, USA). Citral was obtained from Jiangxi Xuesong Natural Medicinal Oil Co. LTD (Jiangxi, China).

*A. alternata* ATCC 66981 was acquired from American Type Culture Collection and cultured on Potato Dextrose Agar (PDA) for 6 days at 25 °C. The spores were washed with 0.1% (*v*/*v*) Tween-80 and adjusted to 1 × 10^5^ spores/mL using a haemocytometer for the following culture.

### 4.2. Antifungal Effects of Citral on Mycelial Growth and Spore Germination

The inhibitory effects of citral were determined by the serial twice dilution method in Potato Dextrose Broth (PDB) medium. The citral was dissolved in ethanol, and then the solution was saved as the stock. The stock solution was diluted to the final concentrations of 0, 0.0625, 0.125, 0.25, 0.5 and 1.0 μL/mL (0, 55.625, 111.25, 222.5, 445 and 890 μg/mL). Quantities of 1 mL of the *A. alternata* spores were inoculated into the medium with the serial concentrations of citral. The strain was then cultured for 6 days at 25 °C, 180 r/min for mycelial weight measurement. The MIC was regarded as the minimum concentration with no mycelium growth of *A. alternata*. An amount of 200 μL of the culture with no mycelia growth was spread onto citral-free PDA and cultured for 6 days at 25 °C. The MFC was determined as the minimum concentration without any mycelium growth on PDA. The fermented mycelium was collected and washed with sterile water to completely remove the medium residues. The mycelia were dried individually by vacuum freeze drying and then weighed.

The trials of fungal spore germination were performed according to a previous method [42,53] with minor modification. The spores were collected and resuspended in sterile double distilled water, and the concentration was adjusted to 1 × 10^5^ spores/mL. One-milliliter aliquots of the spores were mixed with the liquid PDA medium at 45 °C incubation, where citral was added to the final concentrations as described above. The mixture was spread quickly and solidified on Petri dishes. Subsequently, the spores were incubated in the dark at 25 °C. After 24 h of culture, the germinating spores were terminated and dyed with lactophenol cotton blue. The spore germination was judged by the rule that the length of the germ tube must be at least half of the spore diameter, as described by Grbić et al. [42]. At least 200 spores, whether germinated or not, were randomly counted and estimated. Finally, the rates of spore germination were individually calculated.

### 4.3. Determination of Mycotoxin Production

The supernatant of the fermented fungus treated with 0, 1/4MIC and 1/2MIC of citral was separately collected and taken for the mycotoxin measurement. Four milliliters of the acetonitrile were added into 1 mL of the supernatant and mixed thoroughly with a vortex shaker. The mixture was evaporated to dryness by a gentle nitrogen stream at 50 °C. The residue was resolved with 1 mL of acetonitrile–water (30:70, *v*/*v*) and then filtered into a vial with polytetrafluoroethylene (PTFE) membrane. From the vial, 20 μL was injected and assayed by a HPLC-UV/FLD system (Agilent Technologies, Santa Clara, CA, USA) comprising a UV/Visible Detector (258 nm) and FLD Detector (excitation wavelength, 328 nm; emission wavelength, 405 nm), using a reversed phase TC-C18 column (5 μm, 4.6 mm × 250 mm, Agilent, Santa Clara, CA, USA). The column temperature was set at 35 °C, and the flow rate was 1.5 mL/min. The mobile phase was double distilled water and acetonitrile containing 1 mM of oxalic acid. The mycotoxins, AOH and AME, were separated by the mobile phase with a gradient elution rising linearly from 20% to 70% acetonitrile for 15 min at the beginning, maintaining 70% acetonitrile for 1 min, then decreasing to the former 20% acetonitrile for 1.5 min, and finally maintaining 20% acetonitrile for 4.5 min. The mycotoxin concentrations were finally confirmed with UPLC-MS (TQ-S, Waters Micromass, Manchester, UK) under the guidance of Wang et al. [57].

### 4.4. Transcriptome Analysis

The spore suspensions of *A. alternata* (1 × 10^5^ spores/mL) were separately inoculated in PDB with or without 1/2MIC of citral. They were cultured in a shaker incubator at 25 °C, 180 r/min for 6 days. Mycelia were subsequently collected for the transcriptome analysis. RNA extraction, cDNA library construction, RNA-Seq and the following analysis was performed by ShangHai Majorbio Bio-pharm Technology Co., Ltd. (Shanghai, China). Total RNA was extracted by TRIzol reagent (Invitrogen, Life Technologies, Carlsbad, CA, USA) [58]. The mRNA of each sample was enriched by oligo (dT) magnetic beads and then fragmented into the fragmentation buffer. First-strand cDNA was obtained by reverse transcriptase using random hexamers, and then both cDNA strands were synthesized. The purity and concentration of each RNA sample were detected by a NanoDrop 2000 (Thermo Fisher Scientific, Waltham, MA, USA). The integrity of RNA was calculated by 1% agarose gel electrophoresis, and the value of RNA Integrity Number (RIN) was determined by an Agilent 2100 Bioanalyzer using an RNA 6000 Nano kit (Agilent Technologies, Santa Clara, CA, USA). The cDNA library was constructed by the Illumina TruseqTM RNA Sample Preparation kit and sequenced (2 × 150 bp read length) by Illumina HiSeq4000 platforms (San Diego, CA, USA).

The clean data were obtained by removing adaptor sequences, low-quality reads, sequences with 10% higher N rate (N: ambiguous bases information), and over-short sequences of the length from the raw sequenced reads. The clean reads were mapped to the reference genome of *A. alternata* SRC1lrK2f(GCA_001642055) (http://fungi.ensembl.org/Alternaria_alternata_gca_001642055/Info/Index) by the software of Hisat2 [59]. The mapped reads were assembled by StringTie [60,61].

The sequence annotation was analyzed by DIAMOND software [62], searching against the NCBI non-redundant (NR) protein database, Swiss-Prot, and EggNOG database. The classification of GO terms was carried out by BLAST2GO [63]. The protein family was annotated by HMMER3 [64], searching the hidden Markov model (HMM) of the established protein domain against the Pfam database. KEGG pathway annotation was performed by KOBAS 2.1 [65] against the KEGG database.

The read counts were quantified by RSEM software in terms of transcripts per million reads (TPM) [66]. The DEGs were analyzed by DESeq2 [67] and considered as statistical significance of gene expression differences at FDR < 0.05 and the absolute value of Log_2_ FC ≥1. GO enrichment was analyzed in terms of DEGs by Goatools at FDR < 0.05 [68]. The KEGG pathway enrichment analysis of the transcript was conducted by KOBAS 2.1 [65] with respect to DEGs by FDR < 0.05.

### 4.5. Detection of Fungal Ergosterol Content

The fungal ergosterol was extracted and quantified by the minor modified method, under the reference described by Bomfi et al. [26]. Each fungal sample treated with 0 or 1/2MIC of citral was separately homogenized with a glass pestle and completely washed into the solution containing 20 mL of methanol, 5 mL of ethanol and 2.0 g KOH. This was rotated at 25 °C, 250 r/min for 20 min and then incubated for 40 min at 70 °C. Then, this solution was cooled and mixed with 5 mL deionized water. Two milliliters of the supernatant were collected by centrifuging at 10,000 r/min for 10 min. The ergosterol was extracted using an equal volume of n-hexane. The organic phase was evaporated at 50 °C using nitrogen flushing and resolved in methanol. The ergosterol was detected at 282 nm, at the flow rate of 1.5 mL/min, for 15 min using 100% acetonitrile as a mobile phase by a 1260 infinity HPLC-UV system (Agilent Technologies, Santa Clara, CA, USA) after filtration with 0.2 μm polytetrafluoroethylene (PTFE) membrane. The injection volume was 50 μL, and the HPLC separation was performed with a TC-C18 column with a 5 μm particle size (4.6 mm × 250 mm, Agilent, Santa Clara, CA, USA). The ergosterol content was expressed by dividing the fungal mycelia weight of *A. alternata*.

### 4.6. Release of Intracellular Protein

The release of the intracellular soluble protein from *A. alternata* spores was assayed according to the method of Chen et al. [45] with some modification. The spores of *A. alternata* were collected from 6 days culture and washed thrice with sterile double distilled water. The concentration of the released protein was determined after removing the fungal spores and cell debris of *A. alternata*. The spores were centrifuged and then resuspended at the final concentration of 1 × 10^5^ spores/mL in 0.01 M of sterile phosphate buffered solution (PBS, pH 7.2–7.4, Solarbio Life Sciences Inc., Beijing, China) containing different concentrations of citral (0, 1/4MIC, 1/2MIC, MIC and MFC) for 24 h. The leaked protein was determined by bicinchoninic acid (BCA) reagent using a BCA Protein Assay Kit (Solarbio, Beijing, China) and quantified by an EnVision Multilabel Reader (PerkinElmer, Boston, MA, USA) using Bovine Serum Albumin (BSA) as the protein standard.

### 4.7. Scanning Electron Microscopy Analysis

For the microscopic morphology observation, the spores were treated in the medium with or without the MIC of citral. The spores were collected and washed twice with 0.1 M of PBS. Subsequently, they were fixed in 2.5% glutaraldehyde overnight. On the second day, they were centrifuged and washed twice with 0.1 M of PBS. The samples were dehydrated by serial ethanol solutions (30%, 50%, 70%, 80%, 90%) for a period of 15 min. Then, the samples were washed twice in absolute ethanol for 20 min and placed in tertiary butanol twice to completely replace ethanol for 30 min. They were dried through vacuum freeze drying. The samples were mounted on a stub and coated with gold. Finally, the micromorphology observation was individually calculated by Hitachi S-3400 scanning electron microscope at 5 kV accelerating voltage (Tokyo, Japan).

### 4.8. Analysis of Total Antioxidant Capacity and Catalase Activity

The mycelia of *A. alternata* treated with diverse concentrations of citral (0 or 1/2MIC) were collected and washed three times with sterile distilled water. The samples were dried in a vacuum and ground thoroughly using liquid nitrogen. They were separately resuspended in equal volumes of 0.01 M PBS. They were centrifuged, and the supernatants were used for the determination of total antioxidant capacity and catalase activity. The comparison of total antioxidant capacity and catalase activity was calibrated by the protein concentration of each sample. The protein concentration was assayed by a BCA Protein Assay Kit (Solarbio, Beijing, China). All the assays were conducted on an EnVision Multilabel Reader (PerkinElmer, Boston, MA, USA). All the measurements were carried out in triplicate.

Total antioxidant capacity was determined by the T-AOC Assay Kit (Beyotime, Shanghai, China) with the FRAP method. The standard curve was calculated using various concentrations of FeSO_4_ (0, 0.1, 0.25, 0.5, 1.0, 2.5, 5.0 mM). For the FRAP method, the total antioxidant capacity was expressed by the concentration of FeSO_4_ standard solution.

Catalase activity was analyzed by a catalase Assay Kit (Beyotime, Shanghai, China). The supplied H_2_O_2_ in the kit was diluted 100 times, and then the absorbance was detected at 240 nm. The actual concentration (mM) was calculated by the formula of 22.94 × A_240nm_ following the instructions of the kit. The standard curve was measured using the actual various concentrations of H_2_O_2_ (0, 0.625, 1.25, 2.5, 3.75, 5.0 mM). One unit of enzyme activity (1 U) was determined to catalyze the decomposition of 1 μmol of H_2_O_2_ in 1 minute at 25 °C, pH 7.0.

### 4.9. Transcriptional Validation of Biosynthetic Genes Involved in Mycotoxin Production

Total RNA was separately extracted from the treated samples for the following qRT-PCR analysis by an EasyPure Plant RNA Kit (TransGen Biotech, Beijing, China). During the process, the DNA residue was digested by RNase-free DNase I. Total RNA was quantified using a Merinton SMA4000 UV-VIS Spectrophotometer (Ann Arbor, MI, U.S.A) and equally adjusted. The cDNA template was separately synthesized by reverse transcription using the kit of TransScript One-Step gDNA Removal and cDNA Synthesis SuperMix (TransGen Biotech Inc., Beijing, China). The 20 μL quantity of reaction mixture included 10 μL of 2× SYBR Green Master Mix (Applied Biosystems, Foster City, CA, USA), 8.4 μL of double distilled water, 0.6 μL of primer pair (each primer, 10 μM), and 1.0 μL of cDNA as a template. The primers were designed by Primer Premier 6 software based on the sequences of gene transcripts in *A. alternata* (Table S3). The qRT-PCR was then performed by StepOne Plus Real-time PCR systems (Applied Biosystems, Foster City, CA, USA). The relative transcriptional level was separately determined by the 2^−ΔΔt^ method.

### 4.10. Statistical Analyses

All the results were calculated as the mean ± SEM for at least triplicates. The mean differences of the data were compared by analysis of variance (ANOVA) using Tukey’s post hoc test, following the significance at *p* < 0.05 by IBM SPSS statistics 23.0 (IBM Inc., Armonk, NY, USA). The corresponding figures were processed by GraphPad Prism 7.0 (GraphPad Software Inc., San Diego, CA, USA).

## Supplementary Materials

The following are available online at https://www.mdpi.com/2072-6651/11/10/553/s1, Figure S1: Ergosterol content of *A. alternata* in response to different concentrations of citral, Table S1: Statistics of RNA-Seq data from *A. alternata*, Table S2: Identification and functional analysis of gene expression in *A. alternata*, Table S3: Primer sequences designed for quantitative reverse transcription PCR (qRT-PCR) in *A. alternata*.
