# Supplementary material for: Effects of Essential Oil Citral on the Growth, Mycotoxin Biosynthesis and Transcriptomic Profile of Alternaria alternata
Source: Toxins (Basel). 2019 Sep 20;11(10):553. doi: 10.3390/toxins11100553 (PMC6832348; doi:10.3390/toxins11100553)
Supplement: Supplementary file 1 [file toxins-11-00553-s001.zip › toxins-592673-supplementary correction/toxins-592673-supplementary correction.docx]

Supplementary Materials: Effects of Essential Oil Citral on the Growth, Mycotoxin Biosynthesis and Transcriptomic Profile of *Alternaria alternata*

Liuqing Wang, Nan Jiang, Duo Wang and Meng Wang





**Figure S1.** Ergosterol content of *A. alternata* in response to different concentrations of citral.

**Table S1.** Statistics of RNA-Seq data from *A. alternata.*

| **Sample ^a^** | **Raw reads** | **Raw bases** | **Clean reads** | **Clean bases** | **Total mapped** | **Error rate (%)** | **Q20(%)** | **Q30(%)** | **GC content (%)** |
| --- | --- | --- | --- | --- | --- | --- | --- | --- | --- |
| CC_1 | 48,644,700 | 7,345,349,700 | 48,022,338 | 7,124,956,194 | 41,533,625 (86.49%) | 0.0248 | 98.14 | 94.35 | 54.14 |
| CC_2 | 50,827,044 | 7,674,883,644 | 50,225,860 | 7,465,921,432 | 42,975,067 (85.56%) | 0.0247 | 98.17 | 94.43 | 53.88 |
| CC_3 | 49,797,540 | 7,519,428,540 | 49,292,576 | 7,329,237,491 | 42,697,010 (86.62%) | 0.0245 | 98.24 | 94.61 | 53.94 |
| CT_1 | 50,440,054 | 7,616,448,154 | 49,939,542 | 7,443,529,873 | 43,930,630 (87.97%) | 0.0248 | 98.13 | 94.34 | 54.27 |
| CT_2 | 49,206,734 | 7,430,216,834 | 48,683,352 | 7,257,873,079 | 42,851,132 (88.02%) | 0.0244 | 98.27 | 94.68 | 54.23 |
| CT_3 | 52,034,802 | 7,857,255,102 | 51,421,678 | 7,665,472,303 | 46,170,379 (89.79%) | 0.0245 | 98.26 | 94.66 | 54.52 |

^a^ CC: control sample with no citral treatment; CT: sample with citral treatment.

**Table S3.** Primer sequences designed for quantitative reverse transcription PCR (qRT-PCR) in *A. alternata.*

| **Gene** | **Primer** | **Sequence (5’→3’)** | **cDNA length (bp)** |
| --- | --- | --- | --- |
| *β-tubulin* | forward | TCATACTTCGTTGAGTGGAT | 111 |
|  | backward | CTGGATGGAGGTGGAGTT |  |
| *pksI* | forward | TTCACTTCAAGGCGTAGTC | 116 |
|  | backward | CGAAGCTCTTGTCACTAATAC |  |
| *omtI* | forward | GCCTCAGTTGGACTCATC | 117 |
|  | backward | GCAGTTCTCGAAGAAGTGT |  |
| *aohR* | forward | TCCTTATCCTGGACGACAT | 116 |
|  | backward | GAGGTTGATGACGGCTTC |  |
