# Supplementary material for: Effects of Essential Oil Citral on the Growth, Mycotoxin Biosynthesis and Transcriptomic Profile of Alternaria alternata
Source: Toxins (Basel). 2019 Sep 20;11(10):553. doi: 10.3390/toxins11100553 (PMC6832348; doi:10.3390/toxins11100553)
Supplement: Supplementary file 1 [file toxins-11-00553-s001.zip › toxins-592673-supplementary correction/toxins-592673-supplementary correction.pdf]

Supplementary Materials: Effects of Essential Oil Citral on the Growth, Mycotoxin Biosynthesis and Transcriptomic Profile of *Alternaria alternata*

Liuqing Wang, Nan Jiang, Duo Wang and Meng Wang

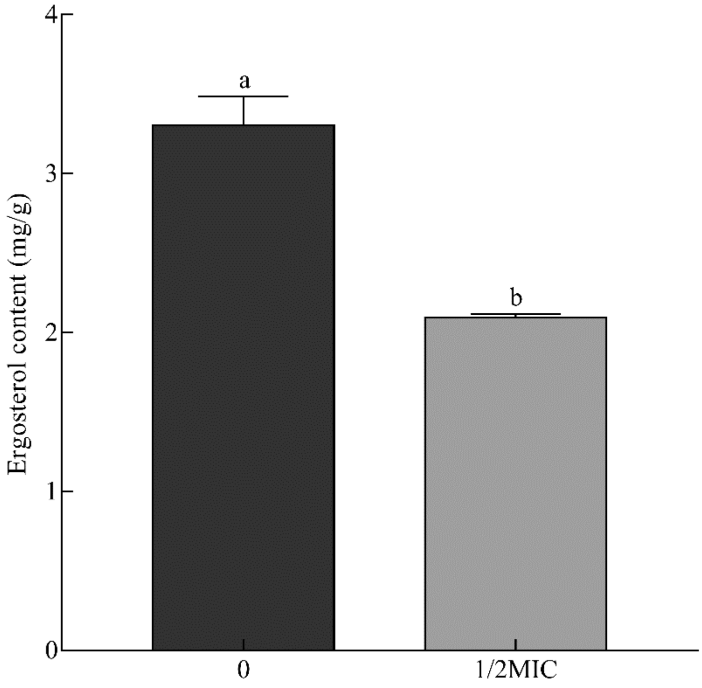

Figure S1. Ergosterol content of *A. alternata* in response to different concentrations of citral.

Table S1. Statistics of RNA-Seq data from *A. alternata.*

Sample [a]	Raw reads	Raw bases	Clean reads	Clean bases	Total mapped	Error rate (%)	Q20(%)	Q30(%)	GC content (%)
CC_1	48,644,700	7,345,349,700	48,022,338	7,124,956,194	41,533,625 (86.49%)	0.0248	98.14	94.35	54.14
CC_2	50,827,044	7,674,883,644	50,225,860	7,465,921,432	42,975,067 (85.56%)	0.0247	98.17	94.43	53.88
CC_3	49,797,540	7,519,428,540	49,292,576	7,329,237,491	42,697,010 (86.62%)	0.0245	98.24	94.61	53.94
CT_1	50,440,054	7,616,448,154	49,939,542	7,443,529,873	43,930,630 (87.97%)	0.0248	98.13	94.34	54.27
CT_2	49,206,734	7,430,216,834	48,683,352	7,257,873,079	42,851,132 (88.02%)	0.0244	98.27	94.68	54.23
CT_3	52,034,802	7,857,255,102	51,421,678	7,665,472,303	46,170,379 (89.79%)	0.0245	98.26	94.66	54.52

[a] CC: control sample with no citral treatment; CT: sample with citral treatment.

Table S3. Primer sequences designed for quantitative reverse transcription PCR (qRT-PCR) in *A. alternata.*

Gene	Primer	Sequence (5′→3′)	cDNA length (bp)
β-tubulin	forward	TCATACTTCGTTGAGTGGAT	111
	backward	CTGGATGGAGGTGGAGTT	
pksI	forward	TTCACTTCAAGGCGTAGTC	116
	backward	CGAAGCTCTTGTCACTAATAC	
omtI	forward	GCCTCAGTTGGACTCATC	117
	backward	GCAGTTCTCGAAGAAGTGT	
aohR	forward	TCCTTATCCTGGACGACAT	116
	backward	GAGGTTGATGACGGCTTC	
